# Validity of using mobile phone surveys to evaluate community health worker program in Mali

**DOI:** 10.1186/s12874-021-01317-7

**Published:** 2021-06-03

**Authors:** Xiaomeng Chen, Diwakar Mohan, Abdoulaye Maïga, Emily Frost, Djeneba Coulibaly, Luay Basil, Birahim Yaguemar Gueye, Mariam Traore Guindo, Assa Sidibé Keita, Haoua Dembelé Keita, Melissa A. Marx

**Affiliations:** 1 Department of International Health, Johns Hopkins Bloomberg School of Public Health, 615 N. Wolfe St, Baltimore, MD 21205 USA; 2 Canadian Red Cross, Ottawa, Canada; 3 Malian Red Cross, Bamako, Mali; 4 National Institute for Research in Public Health (INRSP), Bamako, Mali

**Keywords:** Mali, Implementation strength, Validation, Sensitivity, Specificity, Integrated community case management, Family planning, Community health worker, Mobile phones

## Abstract

**Background:**

The monitoring and evaluation of public health programs based on traditional face-to-face interviews in hard-to-reach and unstable regions present many challenges. Mobile phone-based methods are considered to be an effective alternative, but the validity of mobile phone-based data for assessing implementation strength has not been sufficiently studied yet. Nested within an evaluation project for an integrated community case management (iCCM) and family planning program in Mali, this study aimed to assess the validity of a mobile phone-based health provider survey to measure the implementation strength of this program.

**Methods:**

From July to August 2018, a cross-sectional survey was conducted among the community health workers (ASCs) from six rural districts working with the iCCM and family planning program. ASCs were first reached to complete the mobile phone-based survey; within a week, ASCs were visited in their communities to complete the in-person survey. Both surveys used identical implementation strength tools to collect data on program activities related to iCCM and family planning. Sensitivity and specificity were calculated for each implementation strength indicator collected from the phone-based survey, with the in-person survey as the gold standard. A threshold of ≥ 80% for sensitivity and specificity was considered adequate for evaluation purposes.

**Results:**

Of the 157 ASCs interviewed by mobile phone, 115 (73.2%) were reached in person. Most of the training (2/2 indicators), supervision (2/3), treatment/modern contraceptive supply (9/9), and reporting (3/3) indicators reached the 80% threshold for sensitivity, while only one supervision indicator and one supply indicator reached 80% for specificity. In contrast, most of the stock-out indicators (8/9) reached 80% for specificity, while only two indicators reached the threshold for sensitivity.

**Conclusions:**

The validity of mobile phone-based data was adequate for general training, supervision, and supply indicators for iCCM and family planning. With sufficient mobile phone coverage and reliable mobile network connection, mobile phone-based surveys are useful as an alternative for data collection to assess the implementation strength of general activities in hard-to-reach areas.

**Supplementary Information:**

The online version contains supplementary material available at 10.1186/s12874-021-01317-7.

## Background

Data availability and quality are essential to enable the accountability of health initiatives and programs for assessing the progress of the Sustainable Development Goals (SDGs) [1]. However, data incompleteness and poor data quality remain the major challenges for program evaluations in low- and middle-income countries (LMICs), especially in hard-to-reach areas with logistical or security concerns. The health program evaluations and decision-making processes in LMICs have been using special surveys, such as the Demographic and Health Surveys (DHS) and facility assessments, as the primary data sources. Such surveys consume a considerable amount of workforce, time, and financial resources, in part due to field-related costs. The United Nations Commission on Information and Accountability for Women’s and Children’s Health recommends all countries to use Information and Communication Technologies (ICTs) to obtain “better information for better results” [2]. Mobile phone-based approaches have been demonstrated as helpful and efficient data collection methods in remote and rural areas, enabling more frequent follow-ups [2].

According to the Global System for Mobile Communications Association (GSMA), the percentage of the population in sub-Saharan Africa who subscribe to mobile services increased from 32% in 2012 to 44% in 2018 and is estimated to reach 50% in 2025 [3]. Given the high prevalence of mobile phone ownership in Africa, communicating with survey respondents over their mobile phones may be an alternative data collection method. Such a method is especially relevant for collecting program and health system data from community health workers (CHWs), who are often placed in hard-to-reach areas [4]. Mobile phone-based surveys make it feasible to gather data in remote and high-risk environments, where traditional face-to-face data collection is extremely difficult or dangerous; it also allows rapid response to newly identified needs [5]. Additionally, mobile phone-based data collection methods have been found to be cost-effective in LMICs [6–11]. A prior study in South Africa suggested that mobile phone-based surveys among CHWs can provide reliable results for the supervision of an Integrated Community Case Management (iCCM) program [7]. Studies in Malawi found that data collected by phone interviews had reasonable levels of sensitivity (≥ 80%) in assessing the implementation strength of an iCCM and family planning (FP) program [9, 10]. Nevertheless, the feasibility and validity of mobile phone-based surveys are dependent on the local context and the access to mobile phones. Existing validation studies were based on better-resourced settings, so further assessments of the validity of mobile phone-based data collected in more challenging areas are needed, where climate, transportation, or even conflict restricts access to the field sites [12].

Mali is a low-income country located in sub-Saharan Africa, with one of the highest maternal and child mortality rates and morbidity burden. In 2015, the maternal mortality ratio was estimated to be 587 per 100,000 live births, and the probability of a child dying before reaching the age of five was 115 per 1,000 live births in Mali [13]. However, the accuracy of these data was not perfect. Three of the nine regions in Mali were not covered in the 2012–13 DHS [14] and the 2015 Malaria Indicator Survey (MIS) [15] for security issues. Beginning in 2016, the Canadian Red Cross (CRC), in partnership with the Malian Red Cross (MRC) and the Mali Ministry of Health (MoH), implemented a 4-year Maternal, Newborn and Child Health program in Koulikoro and Sikasso regions. This program purposefully selected villages that were difficult to reach in order to bring health services to these areas. With an ultimate goal of reducing maternal and child mortality and morbidity, the CRC-MRC program recruited community health workers, who were locally called ASCs (Agents de Santé Communautaires), from rural villages; and ASCs were trained to provide FP services and iCCM for children with malaria, diarrhea, acute respiratory infection, and malnutrition. In 2018, an evaluation for this program was conducted in six rural districts (Banamba, Dioila, Kolokani, Koulikoro, Nara, and Sikasso), focusing on the implementation strength of the program. The evaluation used a streamlined protocol for implementation strength assessment (ISA) that was developed for rapid quantitative assessment of the strength of program roll-out by the Real Accountability: Data Analysis for Results (RADAR) program from the Institute of International Programs (IIP) at Johns Hopkins Bloomberg School of Public Health [16]. This protocol has been utilized in Ethiopia and Malawi to assess the strength of iCCM and FP program implementation [9, 10, 17].

The present study is nested within the iCCM and FP evaluation project in Mali. We aimed to assess the validity of implementation strength indicators for iCCM and FP collected from mobile phone-based interviews among ASCs delivering the services in the program districts.

## Methods

### Study design

We leveraged a cross-sectional survey among ASCs recruited in the iCCM and FP program in six rural districts in Mali from July to August 2018. ASCs were first reached by data collectors over mobile phones and completed the phone-based survey. Within a week, the same data collectors visited the ASCs to administer the survey in person. Both interviews used identical implementation strength tools to collect data on program activities related to iCCM and FP. The CRC-MRC program staff provided the study team with a list of mediations, supplies, job aids, and registers that ASCs had been supplied with and should have in stock. These were all asked about by mobile phone or examined in person as part of the survey. We used the indicators collected from the in-person survey as the gold standard to validate the indicators collected from the mobile phone-based survey. In analysis, we paired the responses collected via mobile phone-based interviews with the corresponding responses collected in-person and included only the paired samples for analysis.

### Sample size

Participants in this study were the ASCs working with CRC-MRC and responsible for delivering iCCM and FP services in the program districts. The program implementers provided the sampling frame, including all ASCs from the six program districts who had received training on iCCM and FP and were actively providing services in their communities. All the 441 ASCs on the list were included in the sampling frame, and 300 ASCs were selected for the in-person survey using a stratified random sample proportional to the numbers of ASCs by district for the evaluation project. Having confirmed that all ASCs owned a mobile phone, random sampling proportional to the ASC number was used to select 150 out of the 300 ASCs, and the 150 ASCs were assigned to receive both the mobile phone-based survey and the in-person survey.

**Fig. 1:**
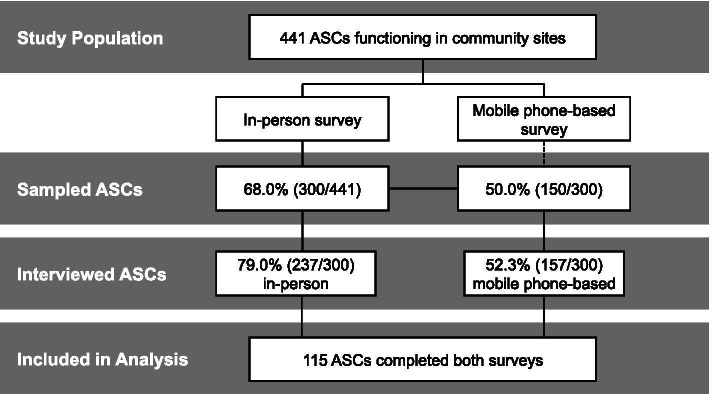
Derivation of the Study Population. Abbreviation: ASC, Agente Sante Communitaire, i.e., community health workers

In practice, 63 out of the 300 ASCs were not reached in-person due to the rainy season (*n* = 50), security issues (*n* = 5), defect phone network (*n* = 2), closure/change of ASC site (*n* = 4), or unavailability (*n* = 2). This study used responses from 115 ASCs who completed both the mobile phone-based and the in-person surveys. This sample size is adequate for sensitivity of 90% and specificity of 90%, with an indicator prevalence at 60% for in-person mode, given the Z statistics set to 1.96 corresponding to the 95% confidence interval [18]. The sampling process and the final sample are described in Fig. 1. According to Buderer’s formula [18], the precision of the sensitivity and specificity estimates for indicators at different prevalence with a type I error of 0.05 is presented in Supplemental Table 1.

### Data collection

The list of ASCs with mobile phone numbers was initially recorded at recruitment and training sessions by the MRC. We then verified the mobile phone numbers by cross-checking with the contact information provided by the ASCs’ supervisors at health facilities, who were the head nurses responsible for the ASCs.

The selected ASCs were first contacted and informed of the study by their supervisors through mobile phones. The supervisors stressed that the evaluation would only be used to improve health services in general but not for individual assessment. The data collectors then reached the ASCs by mobile phone, introduced the study process (including the future in-person interview), and obtained oral informed consent to participate. ASCs who provided oral informed consent were included in the study. The data collectors administered the mobile phone-based survey to the ASCs on the same call. The mobile phone-based survey was required to be completed within three phone calls. If the data collectors did not complete the survey by the third call, no further calls were made, and data were excluded from the analysis. Within a week, the data collectors visited the ASCs in the field to administer the in-person survey using the same assessment tools. Before the start of the in-person survey, data collectors informed about the study again and obtained the written informed consent from the ASCs. During the in-person survey, the ASCs responded to the survey by direct observation from the existing records and stocks; the data collectors also validated the in-person data by inspecting program records, counting the drugs, checking the stock sheets, or examining the paper-based registers, as presented in Supplemental Tables 2 and 3. Any ASCs refusing to participate at any time were no longer contacted for this study, and all data collected up to that point were deleted.

Data were collected from July to August 2018 using an electronic data capture system running Open Data Kit (ODK) on portable Android tablets. Data were entered directly into ODK by the data collectors during the mobile phone-based and in-person interviews. Mobile phone-based data were recorded based on ASCs’ statements, while in-person data were recorded based on the validated statements of the ASCs.

### Survey questionnaire

The survey questionnaire was comprised of three modules: module 1 collected identification information of the ASCs, socio-demographic characteristics (age, sex, socio-cultural groups, marital status, length of living in the village site, and education level), training, supervision, and other work activities; module 2 collected the stocks of drugs, equipment, and materials; and module 3 collected the information recorded on the registers of sick children and FP.

Questions in the training and supervision sections focused on the reception of each activity by the ASC, with the time and content of the activities. Questions in equipment and supplies sections focused on the availability of essential supplies and stock-outs. The list of treatment and modern contraceptive supplies was provided by the CRC-MRC program staff, including: the malaria Rapid Diagnostic Test (RDT), Amoxicillin, artemisinin-based combination therapy (ACT), oral rehydration salts (ORS), zinc, and ready-to-use therapeutic food (RUTF – locally called Plumpy Nut) for iCCM; male condoms, oral contraceptive pills, and injectables for FP. Inventories of supplies were measured by the availabilities of unexpired treatment and modern contraceptive supplies on the day of the interview. The histories of stock-outs were measured by the presence of stock-outs of unexpired treatment and modern contraceptive supplies lasting more than seven consecutive days in the last three months. Regarding the registers for sick child consultations, ASCs were asked about the frequency with which they recorded the date of consultation, name of the child, age of the child, sex of the child, signs/symptoms, diagnosis, and treatment. For the FP registers, the ASCs were asked about the frequency with which they recorded the client’s name, age, and method of choice. In addition, we collected socio-demographic information in both interviews for verification purposes. Both the iCCM and FP questionnaires used in this study were developed by the RADAR program and are available online at https://www.radar-project.org/isaqoc [16].

### Statistical analyses

Providing that the responses from the in-person survey were considered gold standard, the sensitivity and specificity of each indicator were calculated as described below.

The sensitivity of an indicator reported by the mobile phone-based survey was defined as the ability to correctly identify ASCs who reported having the activity or practice. Equation (1) was used for sensitivity computation [19].

1 $$Sensitivity=\frac{True \; positives}{True \; positives+False \; negatives}$$

A true positive meant when an ASC responded “yes” or in an otherwise positive manner to the same question in both the mobile phone-based and the in-person surveys. A false negative occurred when an ASC responded negatively in the mobile phone-based survey while she or he responded positively to the same question in the in-person survey.

The specificity of an indicator reported by the mobile phone-based survey was defined as the ability to correctly identify ASCs who reported not having the activity or practice. Equation (2) was used for specificity computation [19].

2 $$Specificity=\frac{True \; negatives}{True \; negatives+False \; positives}$$

A true negative meant when an ASC responded “no” or in an otherwise negative manner to the same question in both the mobile phone-based and the in-person surveys. A false positive occurred when an ASC responded positively in the mobile phone-based survey while she or he responded negatively to the same question in the in-person survey.

The sensitivity and specificity of each indicator assessed by the mobile phone-based survey were considered adequate for the purpose of ISA if reaching the threshold of ≥ 80%.

To assess the potential selection bias caused by the unavailable in-person visits or mobile phone-based interviews in some ASCs, we examined the differences in characteristics across three groups of ASCs: (1) those who only completed the mobile phone-based survey, (2) those who only completed the in-person survey, and (3) those who completed both surveys. Differences in characteristics by the three groups were tested using Fisher’s exact tests for categorical variables and Kruskal–Wallis tests for non-normally distributed continuous variables. Two-sided p-values < 0.05 were considered statistically significant. All the data analyses were conducted by Stata/SE 14.1.

### Ethical consideration

This study was approved by the Johns Hopkins Bloomberg School of Public Health Institutional Review Board (IRB#00,008,512) and the Faculté de Médecine, de Pharmacie et d’Odontostomatologie Review Board (N°2018/77/CE/FMPOS) in Mali. All participants in this study provided both oral informed consent for the mobile phone-based survey and written informed consent for the in-person survey before data collection.

## Results

### Study population

**Fig. 2:**
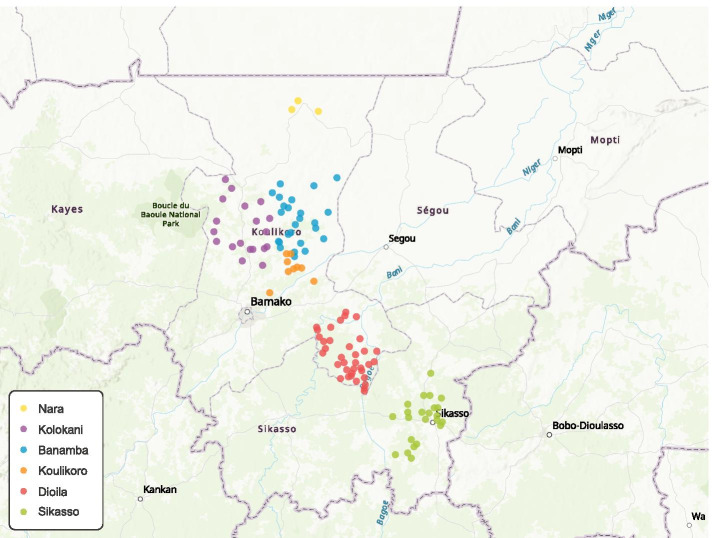
Geographic Distribution of the In-Person Interviews across the Six Districts in Mali. This map was created using ArcGIS® Online by Esri (https://maps.arcgis.com). The geographic data (latitude and longitude) were collected by Open Data Kit (ODK) on the portable Android tablets during in-person interviews

Of the 157 ASCs reached for the mobile phone-based survey, 73.2% (n = 115) were reached in the field for the in-person survey in the six program districts, and these ASCs were included in analyses. The 115 ASCs had a median age of 28 years (IQR: 25–32), and 37.4% were male. The majority of participants were Bambara ethnic group (56.5%), married or engaged (82.6%); 45.2% of participants had primary education, 52.2% had secondary education, and 51.3% had lived in the village for more than three years. The median interval between the mobile phone-based survey and the in-person survey was 4 days, with a total range of 1–11 days. Figure 2 shows the geographic distribution of the 115 in-person interviews.

The distributions of sex, social-cultural group, marital status, education level, and duration of living in the village did not differ between ASCs interviewed for single and both surveys (all p values > 0.2), as shown in Supplemental Table 4. The ASCs reached only by mobile phone were younger than those reached in-person (p = 0.043); fewer ASCs in the two especially hard-to-reach districts (i.e., Nara and Koulikoro) completed the in-person survey (p < 0.001).

### Implementation strength indicators for iCCM

The sensitivity and specificity of the iCCM indicators with observed prevalence were presented in Table 1. According to the in-person survey, 95.7%of ASCs received training in the past two years, and 87.0% received refresher training. Both training indicators had high sensitivity (100.0% and 92.0%) but low specificity (20.0% and 40.0%). For supervision, 82.6% of ASCs were supervised in the past three months, and the indicator had adequate sensitivity (91.6%) and specificity (80.0%). A lower proportion of ASCs (72.2%) reported that they received supervisions that included the component of observation of a sick child consultation within the past three months; the sensitivity for this indicator was 72.3% and the specificity was 40.6%.

**Table 1:** Sensitivity, specificity, and observed prevalence of implementation strength indicators for integrated community case management (iCCM) reported in mobile phone-based survey compared to in-person survey

iCCM indicator	Phone % (n/N)	In-person % (n/N)	Sensitivity % (95% CI)	Specificity % (95% CI)
**Training**				
Proportion of ASCs receiving training in the last 2 years	99.1 (114/115)	95.7 (110/115)	**100.0** (96.7, 100.0)	20.0 (0.5, 71.6)
Proportion of ASCs receiving refresher training	87.8 (101/115)	87.0 (100/115)	**92.0** (84.8, 96.5)	40.0 (16.3, 67.7)
**Supervision**				
Proportion of ASCs supervised in the last 3 months	79.1 (91/115)	82.6 (95/115)	**91.6** (84.1, 96.3)	**80.0** (56.3, 94.3)
Proportion of ASCs receiving supervision which included observation of a sick child consultation in the last 3 months	68.7 (79/115)	72.2 (83/115)	72.3 (61.4, 81.6)	40.6 (23.7, 59.4)
**Treatment supply**				
Proportion of ASCs with a supply of unexpired RDT on the day of the assessment	93.0 (107/115)	93.9 (108/115)	**97.2** (92.1, 99.4)	71.4 (29.0, 96.3)
Proportion of ASCs with a supply of unexpired Amoxicillin tablets/syrup on the day of the assessment	98.3 (113/115)	95.7 (110/115)	**100.0** (96.7, 100.0)	40.0 (5.3, 85.3)
Proportion of ASCs with a supply of unexpired ACT on the day of the assessment	98.3 (113/115)	93.0 (107/115)	**100.0** (96.6, 100.0)	25.0 (3.2, 65.1)
Proportion of ASCs with a supply of unexpired ORS on the day of the assessment	74.8 (86/115)	53.0 (61/115)	**90.2** (79.8, 96.3)	42.6 (29.2, 56.8)
Proportion of ASCs with a supply of unexpired zinc on the day of the assessment	82.6 (95/115)	83.5 (96/115)	**93.8** (86.9, 97.7)	73.7 (48.8, 90.9)
Proportion of ASCs with a supply of unexpired Plumpy Nut on the day of the assessment	47.8 (55/115)	54.8 (63/115)	**81.0** (69.1, 89.8)	**92.3** (81.5, 97.9)
**Treatment stock-outs**				
Proportion of ASCs reporting RDT stock-out that lasted more than 1 consecutive week in the past 3 months	21.7 (25/115)	17.4 (20/115)	75.0 (50.9, 91.3)	**89.5** (81.5, 94.8)
Proportion of ASCs reporting Amoxicillin tablets/syrup stock-out that lasted more than 1 consecutive week in the past 3 months	7.0 (8/115)	24.3 (28/115)	10.7 (2.3, 28.2)	**94.3** (87.1, 98.1)
Proportion of ASCs reporting ACT stock-out that lasted more than 1 consecutive week in the past 3 months	10.4 (12/115)	4.3 (5/115)	40.0 (5.3, 85.3)	**90.9** (83.9, 95.6)
Proportion of ASCs reporting ORS stock-out that lasted more than 1 consecutive week in the past 3 months	16.5 (19/115)	20.9 (24/115)	50.0 (29.1, 70.9)	**92.3** (84.8, 96.9)
Proportion of ASCs reporting zinc stock-out that lasted more than 1 consecutive week in the past 3 months	17.4 (20/115)	15.7 (18/115)	72.2 (46.5, 90.3)	**92.8** (85.7, 97.0)
Proportion of ASCs reporting Plumpy Nut stock-out that lasted more than 1 consecutive week in the past 3 months	53.9 (62/115)	50.4 (58/115)	**86.2** (74.6, 93.9)	78.9 (66.1, 88.6)
**Reporting**				
Proportion of ASCs with complete patient registers for iCCM	98.3 (113/115)	88.7 (102/115)	**98.0** (93.1, 99.8)	0.0 (0.0, 24.7)

Sensitivity and specificity that are considered adequate (≥ 80%) are bolded

n is the frequency of each indicator; N is the total number of ASCs

Abbreviations: *ASC* Agente Sante Communitaire, i.e., community health workers, *iCCM* Integrated community case management, *CI* Confidence interval, *RDT* Rapid diagnostic test for Malaria, *ACT* Artemisinin-based combination therapy, *ORS* Oral rehydration salts

The majority of the treatment supplies were available on the day of the in-person visit (83.5–95.7%), except for ORS (53.0%) and Plumpy Nut (54.8%). All six supply indicators had adequate sensitivity (81.0–100.0%), while only the indicator for Plumpy Nut supply had an adequate level of specificity (92.3%). Most corresponding week-long stock-outs in the past three months were less frequently reported (4.3–24.3%), while the Plumy Nut stock-out was reported by 50.4% of ASCs. Five out of the six stock-out indicators had adequate levels of specificity ranging from 89.5–94.3% while inadequate sensitivity from 10.7–75.0%. Yet, the stock-out indicator for Plumpy Nut had adequate sensitivity (86.2%) while inadequate specificity (78.9%). Most of ASCs (88.7%) were able to produce complete patient registers for iCCM. The sensitivity for this indicator was high (98.0%), but the specificity was 0.0%.

### Implementation strength indicators for FP

The sensitivity and specificity of the FP indicators with observed prevalence were presented in Table 2. According to the in-person survey, most ASCs (79.1%) received supervision on FP in the past three months, with adequate sensitivity of 83.5% but inadequate specificity of 75.0%. Most of ASCs reported available supplies of injectables (87.8%) and oral contraceptive pills (78.3%) on the day of the in-person visit, but fewer ASCs reported the availability of male condoms (59.1%). All three indicators had high sensitivity (92.2–96.0%), while the specificity ranged from 57.1% for injectables to 87.2% for male condoms. Less than one-third of the ASCs reported week-long stock-outs in the past three months for the three modern contraceptive methods; a higher proportion of ASCs reported stock-outs for male condoms (32.2%) than for oral contraceptives (13.9%) or injectables (3.5%). All three stock-out indicators had adequate specificity (82.8–96.4%), while only the indicator for male condom stock-out had adequate sensitivity (91.9%).

**Table 2:** Sensitivity, specificity, and observed prevalence of implementation strength indicators for family planning (FP) reported in mobile phone-based survey compared to in-person survey

FP indicator	Phone % (n/N)	In-person % (n/N)	Sensitivity % (95% CI)	Specificity % (95% CI)
**Supervision**				
Proportion of ASCs supervised in FP in the last 3 months	71.3 (82/115)	79.1 (91/115)	**83.5** (74.3, 90.5)	75.0 (53.3, 90.2)
**Modern contraceptive supply**				
Proportion of ASCs with a supply of unexpired male condoms on the day of the assessment	60.0 (69/115)	59.1 (68/115)	**92.6** (83.7, 97.6)	**87.2** (74.3, 95.2)
Proportion of ASCs with a supply of unexpired oral contraceptive pills on the day of the assessment	79.1 (91/115)	78.3 (90/115)	**92.2** (84.6, 96.8)	68.0 (46.5, 85.1)
Proportion of ASCs with a supply of unexpired injectables on the day of the assessment	89.6 (103/115)	87.8 (101/115)	**96.0** (90.2, 98.9)	57.1 (28.9, 82.3)
**Modern contraceptive stock-outs**				
Proportion of ASCs reporting male condoms stock-out that lasted more than 1 consecutive week in the past 3 months	36.5 (42/115)	32.2 (37/115)	**91.9** (78.1, 98.3)	**89.7** (80.8, 95.5)
Proportion of ASCs reporting oral contraceptive pills stock-out that lasted more than 1 consecutive week in the past 3 months	20.9 (24/115)	13.9 (16/115)	43.8 (19.8, 70.1)	**82.8** (73.9, 89.7)
Proportion of ASCs reporting injectables stock-out that lasted more than 1 consecutive week in the past 3 months	5.2 (6/115)	3.5 (4/115)	50.0 (6.8, 93.2)	**96.4** (91.0, 99.0)
**Reporting**				
Proportion of ASCs with complete patient registers for FP client eligibility	82.6 (95/115)	61.7 (71/115)	**93.0** (84.3, 97.7)	34.1 (20.5, 49.9)
Proportion of ASCs with complete patient registers for FP follow-up	82.6 (95/115)	61.7 (71/115)	**93.0** (84.3, 97.7)	34.1 (20.5, 49.9)

Sensitivity and specificity that are considered adequate (≥ 80%) are bolded

n is the frequency of each indicator; N is the total number of ASCs

Abbreviations: *ASC* Agente Sante Communitaire, i.e., community health workers, *FP* Family planning, *CI* Confidence interval

In terms of reporting indicators, registers for FP client eligibility and registers for FP follow-up had the same prevalence of 61.7%. Both indicators had high sensitivity (93.0%) and low specificity (34.1%).

## Discussion

In this study, the mobile phone-based survey produced adequately accurate data for assessing the implementation strength of the iCCM and FP program in six rural districts in Mali. Specifically, iCCM indicators for training, general supervision, treatment/modern contraceptive supplies, and reporting were adequately sensitive (sensitivity ≥ 80%), while only treatment/modern contraceptive stock-out indicators had adequate specificity (specificity ≥ 80%). Our findings indicated that the validity of mobile phone-based data is limited for relatively detailed information, such as supervision with a particular component (e.g., the observation of case management), availability and stock-outs of specific products, and the completeness of registers.

This study suggested that the ASCs in the hard-to-reach areas in Mali could be successfully reached for mobile phone-based surveys, even during the rainy seasons; yet, it may be more difficult to visit the ASCs in person in these areas. In our study, only 73.2% of ASCs who completed the mobile phone-based survey were successfully reached in person. And this proportion was much lower in Koulikoro and Nara (< 40%) compared to the other districts. There were multiple challenges to visit the ASCs in person in these districts. Nara is the most remote district among the program regions, and the provider sites spread sparsely across the rural areas. There were also safety concerns, such as regional instability and terrorist violence, in Nara. Another challenge for the in-person visits to the ASCs was associated with the rainy season in Mali during the study period, and Koulikoro was the most affected district. Due to the rainy season, 17% of the 300 ASCs who were sampled for the in-person survey were not reached in-person; the interval between the mobile phone-based survey and the in-person survey was extended up to 11 days. Even though the data collectors were trained to validate the data on the basis of the time when the mobile phone-based survey was completed, the prolonged interval might affect the estimations for sensitivity and specificity of the supply and stock-out indicators. Moreover, considering that our study was designed to oversample ASCs in the remote and hard-to-reach areas, the difficulties in data collection in these areas may reflect the difficulties in seeking health services for local people. Therefore, it is pivotal to take the hard-to-reach areas into account when prioritizing equity in health care provision.

There are several potential explanations for the differences in data collected from the phone-based survey compared to the data collected from the in-person survey. First, it is possible that the ASCs did not fully understand the survey questions while on the phone call. A previous study about phone-based interviews found that the respondents did not understand the questions being asked on the phone clearly, especially for the exact definitions of certain documents or technical terms, and this confusion is more likely to occur among respondents with fewer relevant working experiences [10]. Yet, subgroup analyses by ASC characteristics would not have adequate power, given the small sample size of our study. Second, on the data collector side, there might be potential issues in understanding the responses from the ASCs as well. As reported in a previous study in Malawi, data collectors may make errors in reporting or recording data during the phone interviews [9]. This may help explain the lower sensitivity and specificity of indicators involving detailed data such as stock-outs and register completeness. To improve the validity of data collected over phone calls, evaluators may consider conducting a pilot study prior to the formal phone-based survey within the specific cultural and geographic settings so that the pilot results can help inform a better wording of the questions and further reduce potential miscommunications.

To our knowledge, this is the first study designed to assess the validity of the mobile phone-based survey among health care providers in rural districts of Mali, covering two districts where flooding and conflict substantially restricted access to field sites. We extended the prior findings that implementation strength indicators collected via phone survey have adequate validity for program monitoring and evaluation in Malawi [9, 10] into lower-resource and hard-to-reach areas with adequate mobile phone coverage and reliable network connection. The accuracy of mobile phone-based data presented in this study can inform future program monitoring and evaluation plans in hard-to-reach areas.

This study is not without limitations. Initially, even though it is common to use in-person interviews combined with direct observations of the existing records as the gold standard in program evaluations in validation studies [9, 10], the accuracy of the data in the existing records is hard to determine. However, the validated in-person data (based on program records, for example) were the best resource of reference that we could obtain in this study. Furthermore, the sample size in this study was only sufficient to examine the overall validity of the indicators; thus, subgroup analyses, such as district-specific assessments, were not feasible. Additionally, potential selection bias was possible in our sample, as a result of the poor mobile phone connections (mobile phone coverage of 88%) and limited access to the in-person interviews in some areas. Moreover, ASCs were not required to provide feedback regarding the discrepancies of responses from the two surveys, if any. Therefore, we could hardly verify our speculations on the causes of the inadequate sensitivity and specificity.

In summary, the data collected by the mobile phone-based survey in Mali had adequate validity for training, supervision, and supply indicators that only involved general information, yet the validity of the indicators for specific supervision, stock-outs, or complete registers was low. We recommend that local governments and organizations take advantage of the mobile phone-based surveys to monitor and evaluate the implementation strength of programs, especially in hard-to-reach areas.

## Conclusions

Based on the findings from this validation study, mobile phone-based surveys can be used to monitor and evaluate the general implementation strength of public health programs in remote areas, as long as there were sufficient mobile phone coverage, reliable network connection, and an up-to-date contact list of health providers. Further research should be done to improve the validity of mobile phone-based data of specific information, such as certain types of supervision, supplies and stock-outs of multiple products, and patient registers. It is also worthwhile to assess similar phone-based data collection methods in other low-resource settings.

## Supplementary Information

**Additional file 1: Table S1.** Precision of Sensitivity and Specificity Estimates for Indicator at Prevalence of 10-90% with a Type I Error of 0.05. **Table S2.** Definitions and Validation Methods of Implementation Strength Indicators for Integrated Community Case Management (iCCM). **Table S3.** Definitions and Validation Methods of Implementation Strength Indicators for Family Planning (FP). **Table S4.** Characteristics of community health workers who surveyed by phone only and who also surveyed in-person. Statistically significant difference in the distributions at level of α=0.05. *P* values that are <0.05 are bolded.

## Data Availability

The datasets used and analyzed during the current study are available from the corresponding author on reasonable request.

## Abbreviations

ACT: Artemisinin-based combination therapy
ASCs: Agents de santé communautaire (community health workers)
CHW: Community health worker
CRC: Canadian Red Cross
DHS: Demographic and Health Survey
FP: Family planning
GSMA: Global System for Mobile Communications Association
iCCM: Integrated community case management
ICTs: Information and Communication Technologies
IIP: Institute for International Programs
ISA: Implementation strength assessment
LMICs: Low- and middle-income countries
MIS: Malaria Indicator Survey
MoH: Ministry of Health
MRC: Malian Red Cross
ODK: Open Data Kit
ORS: Oral rehydration salts
RADAR: Real Accountability: Data Analysis for Results
RDT: Rapid Diagnostic Test
SDGs: The Sustainable Development Goals
