# Supplementary material for: Validity of using mobile phone surveys to evaluate community health worker program in Mali
Source: BMC Med Res Methodol. 2021 Jun 3;21:115. doi: 10.1186/s12874-021-01317-7 (PMC8176601; doi:10.1186/s12874-021-01317-7)
Supplement: Supplementary file 1 — Additional file 1: Table S1. Precision of Sensitivity and Specificity Estimates for Indicator at Prevalence of 10-90% with a Type I Error of 0.05. Table S2. Definitions and Validation Methods of Implementation Strength Indicators for Integrated Community Case Management (iCCM). Table S3. Definitions and Validation Methods of Implementation Strength Indicators for Family Planning (FP). Table S4. Characteristics of community health workers who surveyed by phone only and who also surveyed in-person. Statistically significant difference in the distributions at level of α=0.05. P values that are <0.05 are bolded. [file 12874_2021_1317_MOESM1_ESM.docx]

**Supplemental Table 1: Precision of Sensitivity and Specificity Estimates for Indicator at Prevalence of 10-90% with a Type I Error of 0.05**

|  | **Indicator Prevalence** | | | | | | | | |
| --- | --- | --- | --- | --- | --- | --- | --- | --- | --- |
|  | **10%** | **20%** | **30%** | **40%** | **50%** | **60%** | **70%** | **80%** | **90%** |
| **Sensitivity** |  |  |  |  |  |  |  |  |  |
| **60%** | 28% | 20% | 16% | 14% | 13% | 12% | 11% | 10% | 9% |
| **65%** | 28% | 19% | 16% | 14% | 12% | 11% | 10% | 10% | 9% |
| **70%** | 26% | 19% | 15% | 13% | 12% | 11% | 10% | 9% | 9% |
| **75%** | 25% | 18% | 14% | 13% | 11% | 10% | 9% | 9% | 8% |
| **80%** | 23% | 16% | 13% | 12% | 10% | 9% | 9% | 8% | 8% |
| **85%** | 21% | 15% | 12% | 10% | 9% | 8% | 8% | 7% | 7% |
| **90%** | 17% | 12% | 10% | 9% | 8% | 7% | 7% | 6% | 6% |
| **95%** | 13% | 9% | 7% | 6% | 6% | 5% | 5% | 4% | 4% |
| **Specificity** |  |  |  |  |  |  |  |  |  |
| **60%** | 9% | 10% | 11% | 12% | 13% | 14% | 16% | 20% | 28% |
| **65%** | 9% | 10% | 10% | 11% | 12% | 14% | 16% | 19% | 28% |
| **70%** | 9% | 9% | 10% | 11% | 12% | 13% | 15% | 19% | 26% |
| **75%** | 8% | 9% | 9% | 10% | 11% | 13% | 14% | 18% | 25% |
| **80%** | 8% | 8% | 9% | 9% | 10% | 12% | 13% | 16% | 23% |
| **85%** | 7% | 7% | 8% | 8% | 9% | 10% | 12% | 15% | 21% |
| **90%** | 6% | 6% | 7% | 7% | 8% | 9% | 10% | 12% | 17% |
| **95%** | 4% | 4% | 5% | 5% | 6% | 6% | 7% | 9% | 13% |

**Supplemental Table 2: Definitions and Validation Methods of Implementation Strength Indicators for Integrated Community Case Management (iCCM)**

| **Indicator** | **Numerator** | **Denominator** | **Validation method** |
| --- | --- | --- | --- |
| **Training** |  |  |  |
| Proportion of ASCs receiving training in the last 2 years | Number of surveyed ASCs trained in iCCM in the last 2 years | Number of ASCs surveyed | Program records |
| Proportion of ASCs receiving refresher training | Number of surveyed ASCs trained in iCCM receiving refresher training | Number of surveyed ASCs trained in iCCM | Program records |
| **Supervision** |  |  |  |
| Proportion of ASCs supervised in the last 3 months | Number of surveyed ASCs supervised in iCCM in the last 3 months | Number of ASCs surveyed | Program records |
| Proportion of ASCs receiving supervision which included observation of a sick child consultation in the last 3 months | Number of surveyed ASCs receiving supervision which included observation of a sick child consultation in the last 3 months | Number of ASCs surveyed | Program records |
| **Treatment Supply** |  |  |  |
| Proportion of ASCs with a supply of unexpired **RDT** on the day of the assessment | Number of surveyed ASCs with a supply of unexpired RDT on the day of the assessment | Number of ASCs surveyed | Counting the drugs |
| Proportion of ASCs with a supply of unexpired **Amoxicillin tablets/syrup** on the day of the assessment | Number of surveyed ASCs in pneumonia districts with a supply of unexpired Amoxicillin tablets/syrup on the day of the assessment | Number of ASCs surveyed | Counting the drugs |
| Proportion of ASCs with a supply of unexpired **ACT** on the day of the assessment | Number of surveyed ASCs with a supply of unexpired ACT on the day of the assessment | Number of ASCs surveyed | Counting the drugs |
| Proportion of ASCs with a supply of unexpired **ORS** on the day of the assessment | Number of surveyed ASCs with a supply of unexpired ORS on the day of the assessment | Number of ASCs surveyed | Counting the drugs |
| Proportion of ASCs with a supply of unexpired **zinc** on the day of the assessment | Number of surveyed ASCs with a supply of unexpired zinc on the day of the assessment | Number of ASCs surveyed | Counting the drugs |
| Proportion of ASCs with a supply of unexpired **Plumpy Nut** on the day of the assessment | Number of surveyed ASCs with a supply of unexpired Plumpy Nut on the day of the assessment | Number of ASCs surveyed | Counting the drugs |
| **Treatment Stock-outs** |  |  |  |
| Proportion of ASCs reporting **RDT** stock-out that lasted more than 1 consecutive week in the past 3 months | Number of surveyed ASCs reporting **RDT** stock-out that lasted more than 1 consecutive week in the past 3 months | Number of ASCs surveyed | Stock sheets |
| Proportion of ASCs reporting **Amoxicillin tablets/syrup** stock-out that lasted more than 1 consecutive week in the past 3 months | Number of surveyed ASCs reporting **Amoxicillin tablets/syrup** stock-out that lasted more than 1 consecutive week in the past 3 months | Number of ASCs surveyed | Stock sheets |
| Proportion of ASCs reporting **ACT** stock-out that lasted more than 1 consecutive week in the past 3 months | Number of surveyed ASCs reporting **ACT** stock-out that lasted more than 1 consecutive week in the past 3 months | Number of ASCs surveyed | Stock sheets |
| Proportion of ASCs reporting **ORS** stock-out that lasted more than 1 consecutive week in the past 3 months | Number of surveyed ASCs reporting **ORS** stock-out that lasted more than 1 consecutive week in the past 3 months | Number of ASCs surveyed | Stock sheets |
| Proportion of ASCs reporting **zinc** stock-out that lasted more than 1 consecutive week in the past 3 months | Number of surveyed ASCs reporting **zinc** stock-out that lasted more than 1 consecutive week in the past 3 months | Number of ASCs surveyed | Stock sheets |
| Proportion of ASCs reporting **Plumpy Nut** stock-out that lasted more than 1 consecutive week in the past 3 months | Number of surveyed ASCs reporting **Plumpy Nut** stock-out that lasted more than 1 consecutive week in the past 3 months | Number of ASCs surveyed | Stock sheets |
| **Reporting** |  |  |  |
| Proportion of ASCs with complete patient registers for iCCM | Number of surveyed ASCs with complete patient registers (date of consultation, name of child, age of child, sex of child, signs and symptoms, classification & treatment) | Number of ASCs surveyed | Paper-based registers |

Abbreviations: ASC, Agente Sante Communitaire, i.e., community health workers; iCCM, integrated community case management; RDT, rapid diagnostic test for Malaria; ACT, artemisinin-based combination therapy; ORS, oral rehydration salts.

**Supplemental Table 3: Definitions and Validation Methods of Implementation Strength Indicators for Family Planning (FP)**

| **Indicator** | **Numerator** | **Denominator** | **Validation method** |
| --- | --- | --- | --- |
| **Supervision** |  |  |  |
| Proportion of ASCs supervised in FP in the last 3 months | Number of ASCs supervised in FP in the last 3 months | Number of ASCs surveyed | Program records |
| **Modern contraceptive supply** |  |  |  |
| Proportion of ASCs with a supply of unexpired **male condoms** on the day of the assessment | Number of surveyed ASCs with a supply of unexpired male condoms on the day of the assessment | Number of ASCs surveyed | Counting the drugs |
| Proportion of ASCs with a supply of unexpired **oral contraceptive pills** on the day of the assessment | Number of surveyed ASCs with a supply of unexpired oral contraceptives on the day of the assessment | Number of ASCs surveyed | Counting the drugs |
| Proportion of ASCs with a supply of unexpired **injectables** on the day of the assessment | Number of surveyed ASCs with a supply of unexpired injectables on the day of the assessment | Number of ASCs surveyed | Counting the drugs |
| **Modern contraceptive stock-outs** |  |  |  |
| Proportion of ASCs reporting **male condoms** stock-out that lasted more than 1 week in the past 3 months | Number of surveyed ASCs reporting **male condoms** stock-out that lasted more than 1 week in the past 3 months | Number of ASCs surveyed | Stock sheets |
| Proportion of ASCs reporting **oral contraceptive pills** stock-out that lasted more than 1 week in the past 3 months | Number of surveyed ASCs reporting **oral contraceptives** stock-out that lasted more than 1 week in the past 3 months | Number of ASCs surveyed | Stock sheets |
| Proportion of ASCs reporting **injectables** stock-out that lasted more than 1 week in the past 3 months | Number of surveyed ASCs reporting **injectables** stock-out that lasted more than 1 week in the past 3 months | Number of ASCs surveyed | Stock sheets |
| **Reporting** |  |  |  |
| Proportion of ASCs with complete patient registers for FP client eligibility | Number of surveyed ASCs with complete patient registers for FP client eligibility (name of woman, age, method of choice) | Number of ASCs surveyed | Paper-based registers |
| Proportion of ASCs with complete patient registers for FP follow-up | Number of surveyed ASCs with complete patient registers for FP follow-up (name of woman, age, method of choice) | Number of ASCs surveyed | Paper-based registers |

Abbreviations: ASC, Agente Sante Communitaire, i.e., community health workers; FP, family planning.

**Supplemental Table 4: Characteristics of community health workers who surveyed by phone only and who also surveyed in-person*.*** Statistically significant difference in the distributions at level of α=0.05. P values that are <0.05 are bolded.

| **Characteristics** | **Mobile phone-based survey only  (N=42)** | **In-person survey only  (N=122)** | **Both surveys (N=115)** | **p value** |
| --- | --- | --- | --- | --- |
| **Age, median (IQR)** | 26.0 (25.0, 28.0) | 28.5 (26.0, 32.0) | 28.0 (25.0, 32.0) | **0.043** |
| **Male sex, %** | 31.0 | 32.8 | 37.4 | 0.66 |
| **Social-cultural group, %** |  |  |  |  |
| Bambara | 42.9 | 47.5 | 56.5 | 0.21 |
| Peuhl | 4.8 | 15.6 | 11.3 |  |
| Senoufo | 19.0 | 11.5 | 9.6 |  |
| Other | 33.3 | 25.4 | 22.6 |  |
| **Marital status, %** |  |  |  |  |
| Married/engaged | 71.4 | 86.1 | 82.6 | 0.28 |
| Single | 28.6 | 12.3 | 16.5 |  |
| Widow | 0.0 | 0.8 | 0.0 |  |
| **Education level, %** |  |  |  |  |
| Primary | 50.0 | 41.8 | 45.2 | 0.92 |
| Secondary | 47.6 | 55.7 | 52.2 |  |
| Higher | 2.4 | 2.5 | 2.6 |  |
| **District, %** |  |  |  |  |
| Banamba | 4.8 | 18.9 | 21.7 | **<0.001** |
| Dioila | 9.5 | 30.3 | 31.3 |  |
| Kolokani | 14.3 | 16.4 | 16.5 |  |
| Koulikoro | 33.3 | 13.9 | 7.8 |  |
| Nara | 11.9 | 3.3 | 2.6 |  |
| Sikasso | 26.2 | 17.2 | 20.0 |  |
| **Living in the village, %** |  |  |  |  |
| Since birth | 7.1 | 4.9 | 4.3 | 0.24 |
| More than 3 years | 31.0 | 49.2 | 51.3 |  |
| Fewer 3 years | 61.9 | 45.9 | 44.3 |  |
